# Learning of food preferences: mechanisms and implications for obesity & metabolic diseases

**DOI:** 10.1038/s41366-021-00894-3

**Published:** 2021-07-06

**Authors:** Hans-Rudolf Berthoud, Christopher D. Morrison, Karen Ackroff, Anthony Sclafani

**Affiliations:** 1 Neurobiology of Nutrition and Metabolism Department, Pennington Biomedical Research Center, Louisiana State University System, Baton Rouge, LA USA; 2 Psychology Department, Brooklyn College of the City University of New York, Brooklyn, NY USA

**Keywords:** Obesity, Obesity

## Abstract

Omnivores, including rodents and humans, compose their diets from a wide variety of potential foods. Beyond the guidance of a few basic orosensory biases such as attraction to sweet and avoidance of bitter, they have limited innate dietary knowledge and must learn to prefer foods based on their flavors and postoral effects. This review focuses on postoral nutrient sensing and signaling as an essential part of the reward system that shapes preferences for the associated flavors of foods. We discuss the extensive array of sensors in the gastrointestinal system and the vagal pathways conveying information about ingested nutrients to the brain. Earlier studies of vagal contributions were limited by nonselective methods that could not easily distinguish the contributions of subsets of vagal afferents. Recent advances in technique have generated substantial new details on sugar- and fat-responsive signaling pathways. We explain methods for conditioning flavor preferences and their use in evaluating gut–brain communication. The SGLT1 intestinal sugar sensor is important in sugar conditioning; the critical sensors for fat are less certain, though GPR40 and 120 fatty acid sensors have been implicated. Ongoing work points to particular vagal pathways to brain reward areas. An implication for obesity treatment is that bariatric surgery may alter vagal function.

## Introduction

The prevalence of obesity and associated metabolic diseases is still increasing globally [1, 2], despite increased awareness and intensive research efforts. It is currently assumed that changes in environment and lifestyle are key drivers in this global pandemic [3]. By providing a background of increased availability of energy-dense foods and physical inactivity there is increased pressure on energy balance regulation that leads to increased adiposity in genetically predisposed individuals [4]. Environmental pressures to overeat are particularly strong and are intricately tied to the modern food industry that promotes the consumption of cheap energy-dense but often nutritionally poor foods beginning in childhood by maximizing palatability and using heavy advertisement [5, 6]. Understanding the physiological mechanisms determining food choice are crucial for the development of behavioral, pharmacological, and even surgical strategies to combat obesity and T2D, and to promote overall healthy eating. Why are we eating what we eat? How does the gut detect ingested nutrients? How does the gut signal nutrient reward to the brain? This review tries to answer at least some of these questions. After a brief description of the many senses and the neurophysiological integrative mechanisms leading to ingestive behavior, we will pay particular attention to gut–brain communication and its role in ingestive behavior and the development of obesity. We will discuss the physiological mechanisms underlying learned nutrient preferences, with special emphasis on sugar and fat preference, for which new mechanisms have recently been proposed.

## The biology of food choice

### Historical background

Given the vital importance of ingestive behavior, its neural control mechanisms are robust, redundant, and evolutionarily conserved. In addition to energy from the three macronutrients, an adequate intake of essential nutrients, vitamins, and minerals is important for survival. All these essential food components are typically mixed in natural and processed foods, and adequate intake of each component is an extremely difficult and complex task for the putative control system. While early nutrition physiologists strongly believed in the ability of animals including humans to solve this complex task without much problem [7, 8], subsequent studies and analyses often failed to support this optimistic assumption (e.g. [9]). Twenty years ago, we edited a book entitled “Neural and Metabolic Control of Macronutrient Intake”, with a collection of over 30 essays by leading scientists laying out their evidence (or lack thereof) for self-regulation of nutrient intake [10]. Lacking much information on the specifics of neural and metabolic controls at that time, the collection of papers was at least able to answer the basic question of whether there is evidence for self-regulation of different nutrients. The general conclusion was that there is a hierarchy in nutrient self-regulation, with good evidence that intake of salt and protein (essential amino acids in particular) are actively defended (hard regulation), but weak evidence for carbohydrates (soft regulation), and little to no evidence for fat (no regulation) [11].

Amino acids cannot be synthesized by the body and are physiologically important, but in contrast, most carbohydrates and lipids can be synthesized internally. Specific putative deficit signals for low-protein (Fibroblast Growth Factor-21, FGF21) and low-salt (aldosterone/angiotensin II), but not for low-carbohydrate or low-fat availability have been identified. A deficit in energy as signaled by low leptin appears to drive intake of all three energy-providing macronutrients equally [12]. However, the absence of specific feedback mechanisms for the intake of carbohydrates and fat does not necessarily mean that there are no mechanisms to detect these nutrients in ingested food and inform other regulatory functions.

### Evidence for self-regulation of protein, carbohydrates, and fat intake

When conducting studies assessing selection between the three macronutrients (protein, carbohydrate, and fat), a common but problematic approach is to provide animals with a single, purified representative of each macronutrient, such as providing casein, sugar, and lard in independent jars within the cage. The weakness of this approach is the potential for the specific sensory properties of the food, such as the powdery dry taste of casein and the greasy taste of lard, to drive selection instead of the nutrient composition itself. To address this approach, multiple representations of the macronutrient should be tested, or more ideally the experiment should include a variety of mixed diets varying in their macronutrient percentage but otherwise nutritionally complete (vitamins and minerals), as in the geometric model of macronutrient selection [13].

Using the geometric model, nutritional state-dependent self-regulation of protein intake has been demonstrated in rats, cats, and insects (for a recent review see: [13]). However, besides liver-derived FGF21 being a driver of protein intake (see Hill et al. for a recent review [14]), details of the neurohormonal signaling mechanisms and pathways underlying the self-regulation of protein intake remain ill-defined despite intensive research efforts (for reviews see: [15–17]).

Carbohydrate and fat intake have recently received much attention from obesity, diabetes, and metabolic disease standpoints. In particular, dietary sugar intake is thought to be a prominent risk factor for these chronic diseases [18, 19]. Behavioral evidence for self-regulation of carbohydrate intake is weak at best [20], and almost absent for fat intake.

### Potential mechanisms for macronutrient choice

**Fig. 1:**
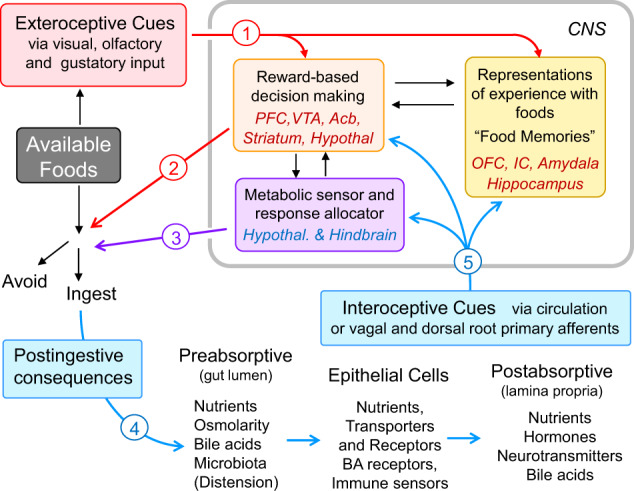
Schematic diagram showing the main flow of information during the task of choosing food. (1) Before ingestion, available foods with their environmental context are perceived through visual, olfactory, and taste cues that may recall memories from previous encounters. (2) Food items found safe and providing positive nutritional signals are selected/preferred over other available foods and ingested. (3) Selection is thereby modulated by the overall nutritional state monitored by the master metabolic sensor in the basomedial hypothalamus. (4) Once accepted and ingested, the chosen food elicits a large number of temporally contingent signals from interaction with components of the alimentary canal, including enteroendocrine cells and neuropod cells. (5) Select signals in the circulation or via primary afferents are used by the brain to initially sustain ingestion (appetition), and later stop ingestion (satiation). They are also used to update existing memories of the selected food, or form new memories. The three general functional brain areas indicated and the specific brain structures included do not necessarily represent the exact neural pathways and systems and rather serve heuristic models. Abbreviations: Acb nucleus accumbens, BA bile acids, IC insular cortex, OFC orbitofrontal cortex, PFC prefrontal cortex, VTA ventral tegmental area (mesolimbic dopamine system).

The basic task of finding a particular nutrient in complex food can be nothing less than the proverbial task of finding a needle in a haystack. Although sight, smell, and taste can contribute important information for finding the needle, they are not necessary. Tasteless mice, knockout mice missing critical taste signaling elements, on normal chow or palatable diets still eat and gain weight, although in some but not all cases significantly less than their wildtype littermates [21–23]. Similarly, it might be an interesting experience having dinner in one of these new restaurants with complete darkness, but the feeling of fullness and satisfaction might be the same even if we eat a little less [24]. In contrast, postoral (post ingestive) detection mechanisms, particularly detection at the level of the intestinal epithelium, where absorption takes place, are crucially important for providing the unconditioned stimulus signaling the arrival of ingested nutrients and leading to fullness, reward, and satisfaction (Fig. 1). As demonstrated in the sham-feeding model with a gastric drainage fistula, a hungry rat will not become satiated in spite of continued ingestion of food for hours. Only placing small amounts of food into the small intestine or systemic administration of cholecystokinin in sham-feeding rats stops food intake and elicits behavioral signs of satiation and satisfaction [25].

Importantly, oral sensory signals such as taste and smell can act as conditioned stimuli determining intake of particular foods through learning. If these signals have reliably predicted the arrival of absorbable and beneficial nutrients (the unconditioned stimulus, US), the food is readily ingested [26, 27]. If the food does not reliably predict the US, then its acceptability will not increase and it may be rejected and the search for a more beneficial food continues (Fig. 1). The reinforcing properties of the US are influenced by the nutritional state, although learning can occur even in food-satiated animals [27]. As shown in Fig. 1, this process is thought to involve a number of pathways and brain areas. Besides the interoceptive and exteroceptive sensory modalities and pathways, areas in the cortex, amygdala, and hippocampus can generate and store representations of experience with specific foods. Together with signals from the hypothalamus and hindbrain reflecting overall nutritional state and from components of the limbic system representing the reward value of specific foods, these “food memories” are then used to make ingestive decisions. However, these central integrative steps subserving food choice are not well understood and are not further considered in this review.

Before looking at experimental paradigms of nutrient-conditioned preferences and recent advances in understanding the mechanisms underlying preference learning for sugar and other macronutrients, we will have a closer look at the organization of gut–brain communication as it pertains to nutritional homeostasis.

## Organization of gut–brain communication subserving nutritional homeostasis

### Mechanosensors

**Fig. 2:**
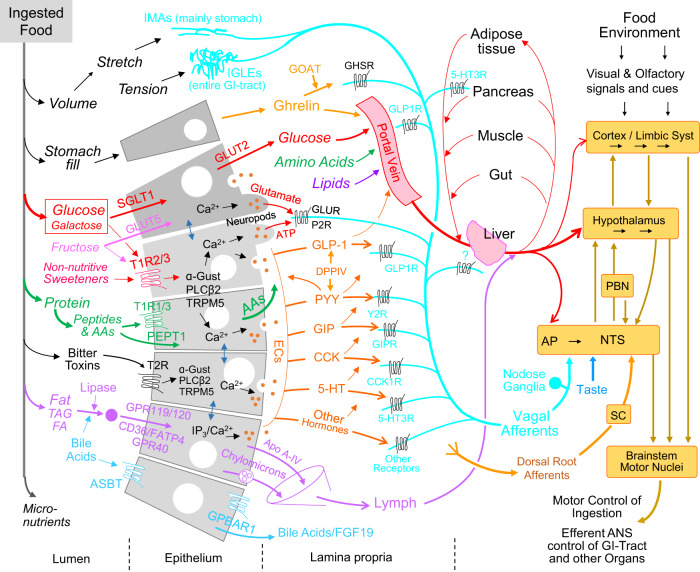
Nutrient signaling in the gastrointestinal tract and its communication pathways to other organs and the brain. The volume and osmotic effects of ingested foods interact with the muscular wall of the alimentary canal and can activate vagal stretch (Intramuscular arrays, IMAs) and tension receptors (Intraganglionic laminar endings, IGLEs). Entry of carbohydrates, proteins, and fats into enterocytes is facilitated by specific transporters localized to the brush border apical membrane. Sugars, amino acids, and lipids are then diffusing into the mucosal lamina propria. Enteroendocrine cells (ECs) represent about 1% of all intestinal epithelial cells that can synthesize and release one or more gut hormones. Once transported into these ECs, carbohydrates, amino acids, and lipids differentially engage intracellular signaling pathways eventually leading to membrane depolarization, increased calcium concentrations, and the release of hormone-containing vesicles into the lamina propria. Some ECs with specialized extensions into the lamina propria (neuropod cells) can release the neurotransmitter glutamate onto vagal afferent nerve terminals bearing glutamate receptors. In addition, sugars and nonnutritive sweeteners are detected by the sweet receptor T1R2/3 and can trigger the synaptic release of ATP in neuropod cells acting on P2R on vagal afferent terminals. Nutrients and hormones in the lamina propria then have access to the bloodstream, mucosal vagal nerve endings, and the lymph system. Nutrients and hormones taken up into the bloodstream (either directly or after transport through the lymphatic system) can interact with vagal sensors in the portal vein or liver and eventually with sensors in all other organs and specific areas of the brain. Crosstalk between different ECs and between ECs and common enterocytes, as well as crosstalk between ECs and the enteric nervous system (ENS) are not shown for simplicity. Also note that innervation of the gut, portal hepatic vein, and liver by dorsal root afferents (DRG), which can also mediate signals to the brain are not shown. Abbreviations: Molecular transduction mechanisms: GLUT2 glucose transporter-2, GLUT5 glucose transporter-5, SGLT1 sodium-glucose transporter-1, T1R2/3 sweet taste receptor, T1R1/3 umami taste receptor, T2R bitter taste receptor, PEPT1 peptide transporter-1, α-Gust α-gustducin, PLC phospholipase C, TRPM5 transient receptor potential cation channel subfamily M member 5, IP_3_ inositol triphosphate, ASBT apical sodium-dependent bile acid transporter, GPBAR1 G protein-coupled bile acid receptor 1. Hormones and enzymes: GLP-1 Glucagon-like peptide-1, PYY peptide YY, GIP Gastric inhibitory peptide, CCK cholecystokinin, 5-HT serotonin, GOAT ghrelin-O-acetyl transferase, DPPIV dipeptidyl peptidase-4, FGF19 fibroblast growth factor 19/15, Apo A-IV apolipoprotein-4. Receptors on vagal afferents: GLP1R GLP-1 receptor, Y2R PYY-2 receptor, GIPR gastric inhibitory peptide receptor, CCK1R cholecystokinin-1 receptor, 5-HT3R serotonin-3 receptor, GHSR growth hormone secretagogue receptor, GLUR glutamate receptor, P2R purinoreceptor. Brain: PBN parabrachial nucleus, AP area postrema, NTS nucleus tractus solitarius, SC spinal cord.

The postoral consequences of foods include interactions with mechanical, chemical, and osmotic sensors (Fig. 2). Vagal stretch receptors (intramuscular arrays, IMAs) are mainly found in the stomach, while vagal tension sensors (intraganglionic laminar endings, IGLEs) are distributed throughout the gastrointestinal tract [28, 29]. Importantly, selective opto- or chemogenetic stimulation of vagal afferent neurons with IGLEs innervating both the stomach and small intestine inhibits 1-h food intake in food-deprived mice by 50% or more [29], suggesting that gastric and intestinal distension significantly contribute to the satiation process. However, because the mechano-sensory signal is blind to the nutritive value of the load, it cannot serve as the US for flavor learning.

### Chemosensors for macronutrients

After emptying from the stomach, nutrients interact with pancreatic juices, bile acids, and microbiota in the small intestinal lumen before traversing the gut epithelial barrier. The epithelial layer consists of several types of cells, including enterocytes, enteroendocrine cells (ECs), and mucin-secreting goblet cells that differentiate from stem cells located in the crypts and are constantly renewed every 3–5 days [30]. ECs are specialized epithelial cells making up less than 1% of the epithelium that function as sensory sentinels, by responding to luminal stimuli and secreting peptide hormones and neurotransmitters [31].

Dietary carbohydrates, proteins, and fats are progressively digested by mastication and salivary enzymes in the mouth, trituration, and acidification in the stomach, and finally by pancreatic juices, bile acids, and microbiota in the lumen of the small intestine, before they are ready for absorption. Glucose and galactose then enter the brush border membrane of enterocytes using almost exclusively the sodium-glucose transporter-1 (SGLT1), while fructose uses the glucose transporter-5 (GLUT5) (for a recent review see [32]). SGLT1 is pivotal for intestinal glucose absorption, as SGLT knockout mice die within two days after weaning when they receive standard starch-based diets [33]. The glucose transporter-2 (GLUT2) is located exclusively at the basolateral membrane at low luminal glucose concentrations, and at both the brush border and basolateral membranes at high luminal glucose concentrations [32]. In addition, nutritive sugars and nonnutritive sweeteners activate the G-protein-coupled sweet taste receptor T1R2/3 expressed in the apical membrane of some ECs [34].

Dietary protein, after hydrolysis by gastric and pancreatic peptidases, is internalized into enterocytes via peptide transporter-1 (PEPT1) linked to the Na^+^/H^+^ exchanger, the calcium-sensing receptor (CaSR), and the recently deorphanized G protein-coupled receptor GPRC6A [34, 35]. Small peptides and individual amino acids are then transported by peptide and amino acid transporters across the basolateral membrane into the lamina propria. In addition, certain amino acids such as glutamate activate the G-protein-coupled umami taste receptor T1R1/3 [34].

Dietary fats, after being emulsified and processed into mixed micelles through the action of lipases and bile acids, are transported into enterocytes by (1) the fatty acid transporter-4 (FATP4), (2) fatty acid translocase (CD36) with the help of membrane-bound (FABP_m_) and cytoplasmic (FABP_c_) fatty acid-binding proteins, and (3) the Nieman-Pick C1 like 1 protein (NPC1L1) [36]. Long- and medium-chain containing triglycerides and cholesterol are then assembled into chylomicrons and exported through the basolateral membrane where they are transported by the lymphatic system to the general circulation, while short-chain fatty acids (SCFAs) are freely diffusing through enterocytes to reach the bloodstream through the hepatic-portal vein [36].

Individual enteroendocrine cells can produce different combinations and quantities of peptide hormones and are sprinkled in different proportions over the length of the gastrointestinal tract. CCK and GIP cells are enriched in the upper small intestine, GLP-1 and PYY in the lower small intestine and colon, and ghrelin in the stomach [37]. Importantly, specific intracellular signaling mechanisms involving ion channels, membrane depolarization, and intracellular calcium, link nutrient absorption to hormone release, whereby each macronutrient elicits its specific fingerprint of gut hormones released [37] (Fig. 2). Given the scarcity of enteroendocrine cells among the many absorptive enterocytes, paracrine crosstalk between common enterocytes and enteroendocrine cells as well as between enteroendocrine cells is important [38]. Thus, enteroendocrine cells are sentinels transducing bulk macronutrient absorption into the information available for the gut itself and for all other organs (Fig. 2).

### Neural signaling pathways to the brain

The gastrointestinal tract is heavily innervated by both vagal and dorsal root afferents. Dorsal root afferents are generally thought to mediate pain rather than normal physiological signals [39], but a role in nutrient homeostasis is not excluded. Spinal primary afferent neurons with cell bodies in dorsal root ganglia innervate the entire gastrointestinal tract and associated glands, and their total number compares well with the number of vagal subdiaphragmatic afferents [40]. Single spinal visceral afferents distribute over many segments [41], thus contributing to homeostatic regulation of a wide range of organs. Furthermore, they gain easy access to most brain areas through the spino-solitary, spino-parabrachial, spino-hypothalamic, and other tracts and therefore have the potential to affect the same brain areas that are affected by vagal afferents.

Here we focus on vagal afferents, for which there is rich literature describing their role in nutrient homeostasis and ingestive behavior. We have already introduced vagal afferent innervation of the external muscle layers of stomach and intestines by IMA and IGLE mechanosensors and their ability to modulate food intake. However, vagal afferents innervating the mucosa throughout the gastrointestinal tract are in a much better position to sense the chemical milieu in the lamina propria, as their terminals are in close contact with freshly absorbed nutrients [42, 43] and ECs with their secretory products [44, 45]. There is plenty of older literature, from before the discovery of most gut hormones, suggesting that vagal afferents are sensitive to a variety of nutrients, including glucose, amino acids, and fatty acids [46–50]. Later, expression of many gut hormone receptors by vagal afferents innervating the gut, and at least some evidence for their role in ingestive behavior was reported. After the early discovery of CCK, the potential role of CCK and its CCKA-receptor on vagal afferents in the process of satiation was of most interest [42, 43, 51–53]. More recently, interest shifted to the role of GLP-1 released from intestinal L-cells and the GLP-1 receptor expressed by vagal afferents in satiation [54–56].

However, sub-optimal methodology in many of these earlier studies often prevented clear conclusions to be drawn. Perhaps the major problem was an inability to manipulate and visualize functionally specific populations of vagal afferents. Vagotomies were typically non-specific, not only regarding afferent subtype and specific tissue/organ innervated, but also regarding afferent vs. efferent. Visualization of receptors was typically limited to immunohistochemistry of vagal afferent neuronal cell bodies in the nodose ganglia, without knowing their specific innervation targets. This is exemplified by experiments in rodents surgically interrupting the common hepatic branch dividing from the left subdiaphragmatic vagal trunk. The rat common hepatic vagal branch contains both afferents and efferents (and even some non-vagal nerve fibers [57], and projects primarily to the proximal duodenum, pylorus, and pancreas via the gastroduodenal artery. It also innervates the portal hepatic vein, and only a small fraction actually innervates the liver itself along the hepatic artery [58]. Therefore, this complicates the interpretation of the functional effects of common hepatic branch vagotomy, particularly when looking at longer-term effects.

Specific labeling and manipulation of sub-populations of vagal afferents by genetics-based tools is the most significant advance for understanding their role in nutritional homeostasis [29, 59–65]. Two studies, in particular, reported molecular maps of target-specific vagal sensory neurons using single-cell RNA sequencing [29, 64]. This allowed the generation of separate *Cre*-mouse lines and identification of their unique morphologies and innervation patterns in the gastrointestinal tract [29], confirming the presence of the three distinct sensory terminal architectures, namely IMAs, IGLEs, and mucosal endings, previously described after nonselective anterograde tracing with DiI in the rat (as summarized in [28]). In addition, however, the genetic approach allows selective manipulation (acute and chronic stimulation and inhibition) of such specific populations of vagal sensory neurons [29, 61].

Besides releasing gut hormones, some specialized enteroendocrine cells (neuropod cells) penetrate the basolateral membrane and can release neurotransmitters directly on vagal afferent nerve terminals that are synaptically opposed [66]. More recently, these neuropod cells have been demonstrated to mediate the SGLT1-dependent glucose-signal rapidly to vagal afferents through glutamatergic signaling [67–69]. Such direct synaptic connections allow for very rapid signaling to the brain and together with the viscerotopic organization of vagal afferents have the potential to inform the brain what is absorbed at a given location on a second-by-second basis.

### Humoral signaling

Given that the focus of this review is on nutrient-conditioned preferences and that much recent work implicates neural pathways, our discussion of humoral mediation is limited to a few essential points. For more comprehensive reviews on humoral gut–brain signaling relevant to obesity and metabolic disease see e.g., [70]. Besides signaling through primary afferents, nutrients and hormones can also signal to the brain via blood circulation. Once released into the lamina propria they are taken up by mucosal capillaries to reach the hepatic-portal vein and eventually all other organs including the brain. Some gut hormones such as GLP-1, PYY, and ghrelin, are subject to modifications by peptidases and other enzymes, which can greatly reduce or enhance their binding to specific receptors. Concentrations of specific nutrients and hormones are significantly higher in hepatic-portal blood compared to general arterial blood concentrations. Chylomicrons and hormones such as ApoAIV and GLP-1 are also transported by the lymph system, which bypasses the hepatic-portal vein and liver, to enter the general circulation via the subclavian vein [36].

In the brain, nutrients and hormones can more or less affect neurons and glia depending on the permeability of the blood–brain barrier. Areas without or with a weak blood–brain barrier such as the area postrema in the hindbrain, and the median eminence in the basomedial hypothalamus are most strongly affected, but hormones and nutrients can affect most other brain areas if adequate transport systems exist. Hormones and other humoral factors such as leptin, insulin, and FGF21 secreted by these other organs are clearly important for overall nutritional homeostasis, by interacting with humoral and neural signals from the gut at many levels.

In contrast to the fast, high fidelity neural connections, humoral signaling is slower and generally conveys little viscerotopic information. On the other hand, humoral signals have the potential to act in a more sustained and integrative fashion.

## Experimental paradigms for food preference learning

A broad question, which has been answered in increasing detail in recent years, concerns which of the gut sensing and signaling mechanisms described in the previous sections are crucial for the development of food preferences. This section introduces the techniques used to train and measure preferences in laboratory rodents.

**Fig. 3:**
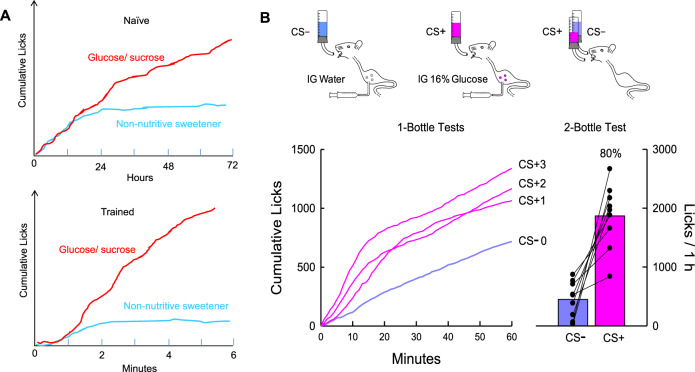
Nutrient-conditioned flavor preferences. **A** Naïve mice given two-bottle access to “isosweet” nutritive sugars (glucose or sucrose) and nonnutritive sweeteners (sucralose, AceK) take 24 h or more to develop a preference for the sugar. Once trained, a sugar preference is expressed in less than 2 min [65, 68, 136]. **B** Naïve mice given one-bottle access (1 h/day) to a CS+ flavored saccharin solution paired with IG 16% glucose infusion increase their licking response within 10 min in the first test session (CS+1) compared to prior sessions with a CS- flavor paired with IG water (CS-0). In subsequent one-bottle CS+ sessions licking is increased from the very first min. In two-bottle tests all mice licked more for the CS+ than CS−; 80% CS+ preference. Because mice were not infused in 2-bottle tests they licked much more than in one-bottle tests [94].

Animals learn to associate the flavor of food, that is, its taste, smell, texture, and other oral chemesthetic cues with the food’s postoral (post ingestive) consequences [26, 27, 85]. This learning can occur with short- or long-term sessions (30 min–24 h) and under food sated or restricted states. In the laboratory, the “food” is often a flavored nonnutritive solution (or gel) with postoral consequences manipulated by the experimenter. The outcome of this learning is typically expressed in subsequent encounters with the food in choice (e.g., two-bottle test) or no-choice (one-bottle test) situations. If the food contains toxins or poorly digested nutrients (e.g., lactose) that produced gastrointestinal distress, animals rapidly learn to avoid its flavor. Conditioned flavor aversions are well documented as reviewed elsewhere [27, 72, 73]. Of interest here are flavors that are associated with positive reinforcing consequences [27]. In this case, animals may learn to prefer the flavored solution (conditioned flavor preference) as evidenced by their preferential intake in choice tests and may also increase their absolute intake of the flavored solution (conditioned flavor acceptance) (Fig. 3). Total intakes may not increase with concentrated nutrient sources which limit intake although initial rates of ingestion and/or meal sizes may be enhanced [74]. This process, in which the ingestion/absorption of nutrients promotes positive associations that increase preference is termed appetition, and thus postoral cues that increase preference and/or acceptance are referred to as ‘appetition’ cues to distinguish them from ‘satiation’ cues that decrease intake [75].

A simple procedure to study flavor-nutrient learning is to train animals on alternate days to consume a novel flavor (the conditioned stimulus, CS+, e.g., grape) mixed in a nutrient solution (the unconditioned stimulus, US, e.g., sucrose) and a different flavor (the CS−, e.g., cherry) mixed in water and then assess the conditioned preference/acceptance in subsequent choice tests with the CS+ and CS− flavors presented in water. A potential problem with this paradigm, however, is that the animal may acquire a CS+ flavor preference based on its association with the palatable flavor of the nutrient (e.g., sweet taste) rather than (or in addition to) the nutrient’s postoral actions. Flavor-flavor learning is demonstrated by the learned preference for a CS+ flavor mixed into a nonnutritive sweet solution (e.g., saccharin, sucralose) [27]. To eliminate this flavor-flavor association, animals can be trained with the CS+ flavor added to a sugar solution and the CS− flavor added to a nonnutritive solution matched in palatability to the sugar [76, 77]. Any resulting CS+ preference can thereby be attributed to the postoral actions of the sugar rather than its sweet taste. In one variation of this procedure, animals are trained to consume sugar and nonnutritive sweetener solutions (without added flavors) with the nonnutritive solution being matched or even more palatable than the sugar solution [65, 78] (Fig. 3A). If animals develop preferences for the sugar (which is both the CS+ and US) over the nonnutritive sweetener (CS−) after training, this preference is indicative of postoral sugar conditioning. This type of learning is possible because even if the sugars and nonnutritive sweeteners are “isosweet”, they differ in other flavor characteristics that allow animals to discriminate their flavors. Thus, postoral sugar conditioning can enhance the innately attractive sweet taste of sugar itself as well as for any associated flavors (e.g., the flavor of a sugar-rich mango).

An alternative procedure to investigate flavor-nutrient learning is to train animals to drink differently flavored solutions of similar palatability (e.g., both unsweetened or saccharin-sweetened fruit flavors) but with the CS+ flavor paired with intragastric (IG) nutrient infusions and the CS− paired with IG water infusions [26, 27] (Fig. 3B). Flavor preferences can be conditioned by IG infusions of complete liquid diets or individual macronutrients (carbohydrate, fat, protein). This conditioning method is very potent in that it (a) can convert innate aversions to bitter or sour tastes to strong preference and (b) produces long-lasting preferences that are resistant to forgetting or extinction [27, 85]. Another method for evaluating the reinforcing actions of nutrients involves pairing a place (e.g., distinctive chamber) or sipper tube position with the consumption of a nutritive substance (e.g., sucrose solution) [79, 80]. Unlike the case of conditioned flavor preferences, the resistance to extinction of conditioned place/position preferences over several trials has not been established. More recently, postoral nutrient reinforcement has been evaluated in mice by using self-administration procedures in which an operant response (licking unflavored water or a dry sipper tube, lever pressing) is reinforced by IG nutrient infusions (e.g., sugar, fat) [71, 81–83]. As discussed below, a new development in the study of food preference learning is the use of opto/chemogenetic approaches to target-specific neurons activated by postoral nutrients to condition flavor preferences or block the expression of previously learned preferences [65, 68, 84].

## Mechanisms for sugar-conditioned preferences

The nutrient conditioning actions of carbohydrates are extensively documented using various sugars, maltodextrins, or starches [27]. Rats and mice trained in alternate daily sessions (30 min–24 h) to drink a CS+ flavored solution paired with concurrent IG infusions of 8–32% glucose-based carbohydrates (glucose, sucrose, maltose, maltodextrin) and a CS− flavor paired with IG water infusions subsequently displayed a significant (70–90%) preference for the CS+ over the CS− flavor in two-choice tests [27, 85] (Fig. 3). Carbohydrate conditioned preferences have been considered to be a form of “flavor-calorie” learning, but isocaloric carbohydrates can differ substantially in their effectiveness to condition flavor preferences. In particular, in rats and some mouse strains (FVB/N) IG fructose infusions condition much weaker flavor preferences than do isocaloric glucose infusions and in some mouse strains (e.g., C57BL/6, B6) IG fructose is completely ineffective [74, 86–88].

### Transduction site of postoral sugar signal

**Fig. 4:**
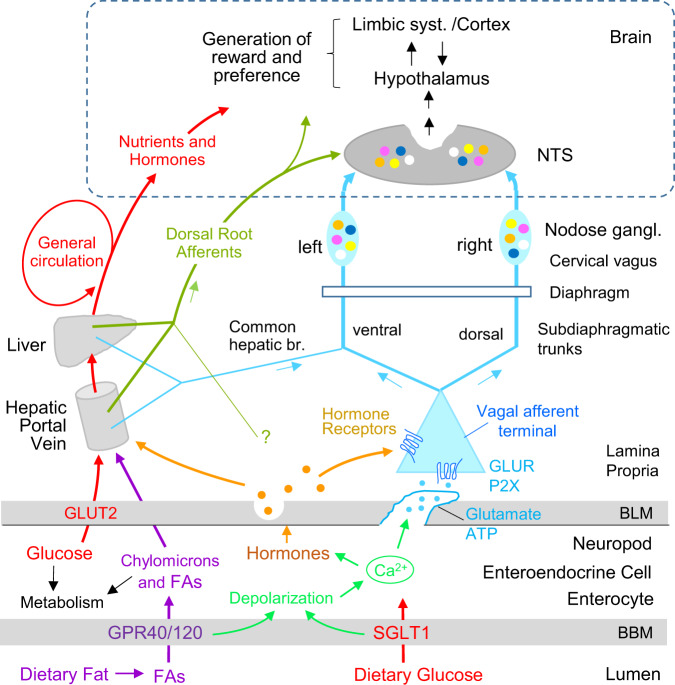
Proposed gut–brain pathways mediating postoral sugar and fat appetition in mice. (1) SGLT1-mediated glucose transport across the brush border membrane leads to enterocyte depolarization and the release of glutamate from neuropod cells reaching into the lamina propria. The synaptically released glutamate excites glutamate receptors located on sensory nerve terminals originating from unknown vagal afferent neuron populations in both the left and right nodose ganglia and projecting through both left and right cervical vagus [68]. (2) Glucose activates via SGLT1 a selective population of vagal afferent neurons and in turn a selective population of proenkephalin-expressing neurons in the left and right NTS [65]. (3) Glucose metabolism can influence brain reward circuitries by an unknown metabolic sensor and pathway [96]. (4) After absorption and reaching the hepatic-portal vein and liver, glucose activates the mesolimbic dopamine system by acting in an unknown fashion on sensory terminals of vagal afferent fibers passing through the common hepatic branch associated with the left cervical vagus [83]. (Note that these authors speculate that the postoral sucrose may act on neuropod cells or hepatic-portal sensors, admit that there must be pathways in addition to the hepatic vagus; and their outcome behavior is operant sugar seeking) (5) The presence of intestinal glucose is signaled in an SGLT1-dependent fashion via dorsal root afferent neurons passing through the celiac ganglia to inhibit hypothalamic AgRP neurons [103]. (Note that there was no preference testing in this study). Inhibiting AgRP neurons conditions flavor preferences [137]. (6) Fatty acids (FA) derived from dietary fat acting in part on intestinal GPR40 and GPR120 sensors signal brain reward circuits via undefined pathways to condition CS+ flavor preferences and promote fat-seeking behavior [112]. (7) Dietary fat acting on unspecified intestinal sensors activate brain reward systems via CCK-sensitive vagal afferent fibers passing through the right nodose ganglion to condition relative preferences for dilute or concentrated fat emulsions and promote operant fat-seeking behavior [84]. (8) Dietary fat acting on unspecified intestinal sensors via vagal afferent neurons to inhibit hypothalamic AgRP neurons [103]. Note that studies in rats indicate that the upper small intestine is partially innervated by vagal fibers traveling in all the anterior and posterior celiac, the anterior and posterior gastric, as well as the gastroduodenal branch dividing from the common hepatic branch [28].

Information on the site(s) of action for postoral carbohydrate conditioning is provided by results obtained with different postoral infusions. In rats, (a) IG glucose infusions conditioned flavor preferences only when the sugar was allowed to empty into the intestinal tract [89] (b) glucose infused in the duodenum or jejunum, but not the ileum, conditioned flavor preferences [90]; and (c) glucose infusions into the hepatic-portal vein failed to condition preferences for a nonnutritive CS+ solution [90]. These results implicate the upper intestinal tract as a critical site of action for glucose sensing [85] (Fig. 4). Hepatic-portal glucose infusions conditioned a preference for a CS+ flavored chow that itself provided intestinal nutrient stimulation [91], suggesting that portal glucose is an effective conditioning stimulus when combined with preabsorptive nutrient stimulation. Consistent with this interpretation, portal glucose infusions conditioned preferences for flavored glucose but not for flavored saccharin solutions [90]. Hepatic-portal glucose infusions, however, conditioned a sipper tube side preference and increased dopamine release in the nucleus accumbens which is critical for preference conditioning in rats [92]. Thus, postabsorptive glucose alone supports at least some forms of preference conditioning.

Sweet taste signaling proteins (T1R2, T1R3, gustducin, TRPM5) are expressed in intestinal cells which suggests that intestinal “sweet” sensing could mediate postoral sugar conditioning (Fig. 2). However, this is not supported by the findings that IG infusions of sweet receptor ligands fructose and sucralose do not support flavor conditioning in B6 mice [93, 94]. Furthermore, IG sugar infusions condition strong flavor preferences in knockout (KO) mice lacking T1R3, gustducin, or TRPM5 [85]. Rather than intestinal sweet receptors, glucose-specific sensors/ transporters (SGLT1, SGLT3, and GLUT2) are implicated in postoral sugar conditioning. In B6 mice, IG infusions of α-methyl-D-glucopyranoside (MDG), a non-metabolizable glucose analog that binds to SGLT1 and SGLT3, conditioned a CS+ flavor preference that was blocked by co-infusions of the SGLT1/3 inhibitor phloridzin [94]. IG glucose conditioning was blocked when the infusion included both SGLT1/3 and GLUT2 inhibitors, implicating GLUT2 in glucose conditioning. However, the genetic deletion of SGLT1 was sufficient to block IG conditioning by MDG and glucose [95]. Note that glucose conditions stronger preferences than MDG, which may be due to the ability of postabsorptive glucose but not MDG to promote striatal dopamine release [94, 96]. In addition, the accumulation of the non-metabolizable MDG in the body may generate inhibitory signals that suppress conditioning. Nevertheless, the differential conditioning actions of glucose, fructose and non-metabolizable MDG are remarkable and indicate that “the signaling system recognizes the sugar molecule itself rather than its caloric content or metabolic products” [65, 94].

### Gut–brain pathway for unconditioned sugar signal

The gut–brain pathway(s) that mediate postoral glucose preference conditioning is not fully understood (Fig. 4). Several studies reported that surgical transection of the subdiaphragmatic vagal trunks (SDV) or subdiaphragmatic deafferentation (SDA) did not prevent glucose-conditioned flavor preferences [97–100]. However, other recent findings implicate a central role for vagal afferents. In particular, intestinal infusions of glucose, sucrose, and MDG, but not fructose were found to act on intestinal neuropod cells and rapidly stimulate vagal afferents via glutamatergic synaptic connections [67, 68] (Fig. 4). In addition, optogenetically silencing the neuropod or pharmacologically inhibiting the glutamatergic vagal synapse blocked the expression of a learned preference for sucrose over sucralose [68]. Tan et al. [65] further reported that intestinal infusions of glucose and MDG but not the nonnutritive sweetener acesulfame K (AceK) activated a bilateral subset of proenkephalin-expressing neurons in the caudal nucleus of the solitary tract (cNTS). The cNTS response was blocked by acute bilateral surgical cervical vagotomy. In 48-h, two-bottle choice tests, B6 mice initially consumed similar amounts of 600 mM glucose and 30 mM AceK solutions but developed a strong glucose preference by the end of the test (Fig. 3). Similar preference changes were observed with MDG vs. AceK but not with fructose vs. AceK, consistent with differential flavor conditioning actions of IG glucose, MDG, and fructose [94]. Evidence that the intestinal-vagal-cNTS circuit activated by intestinal glucose and MDG is responsible for the preference conditioning effects of these sugars is indicated by the findings that (a) selective silencing of neurochemically-defined vagal sensory neurons in the nodose ganglia blocked the development of a preference for glucose over AceK and (b) selective silencing of the proenkephalin-expressing cNTS neurons activated by intestinal glucose also blocked development of a preference for glucose [65]. Furthermore, silencing cNTS neurons prevented the overconsumption of glucose, relative to AceK, driven by the sugar’s postoral actions.

While the above findings provide compelling evidence that the intestinal-vagal-cNTS circuit mediates the glucose preference conditioning, they do not account for the failure of surgical SDV or SDA procedures to block glucose-conditioned preferences [97–99]. However, it should not be surprising that these very nonselective vagotomies led to misleading outcomes, particularly in chronic situations. Because these crude vagotomies eliminate a great number of vagal fibers with different functionalities, they likely lead to adaptive changes in the bidirectional signaling between the gut and the brain over time. In addition, they may spare critical afferent vagal fibers that are deactivated by optogenetic or neurochemical silencing of neuropod cell signaling or nodose afferents [101]. Alternatively, there may be afferent fiber regeneration after surgical SDV or SDA vagotomy but not after neurochemical nodose vagotomy. Given the finding that acute surgical cervical vagotomy blocked intestinal glucose activation of cNTS neurons [65], it would be most informative to determine if intestinal glucose activates cNTS neurons in animals with acute or chronic SDV or SDA surgery.

Another consideration is the sufficiency of sugar-induced activation of vagal afferents to condition flavor preferences. The differential vagal activation effects of glucose, MDG and fructose [65] are consistent with the differential flavor conditioning effects observed with IG infusions of these sugars [85, 86, 94]. However, intestinal infusions of galactose and non-metabolizable 3-O-methyl-d-glucose (OMG) were similar to glucose and MDG in stimulating vagal nerve activity [65] but IG galactose and OMG were much less effective than glucose and MDG in conditioning CS+ flavor preferences [87, 94]. Because glucose and MDG, unlike galactose and OMG, are ligands for the glucose sensor SGLT3 as well as SGLT1, perhaps both SGLT sensors mediate preference conditioning, although SGLT3 involvement remains uncertain [95]. Alternatively, galactose and OMG may have postabsorptive inhibitory actions that interfere with flavor conditioning [95]. Whatever the reason, the similar vagal activation patterns observed with these four sugars do not correlate with their flavor conditioning effects.

Even in the absence of unique flavor cues, postoral sugar sensing can modulate consumatory and appetitive behaviors to obtain sugars. This was demonstrated by the effectiveness of IG sucrose and glucose infusions to reinforce operant licking of an empty sipper tube in B6 mice [81, 82]. In contrast, B6 mice do not maintain operant licking for IG fructose infusions, which is consistent with the failure of IG fructose to condition flavor preferences [81]. More recently, Fernandes et al. [83] reported that oral sucrose and IG sucrose both reinforced operant lever pressing in B6 mice. A critical role for brain dopamine circuits in mediating lever pressing for IG sucrose infusions was revealed by the findings that (a) IG sucrose infusions activated dopamine neurons in the VTA and (b) KO mice with impaired VTA DA neuron function were deficient in their lever pressing for sucrose rewards. The involvement of the hepatic branch of the left vagus nerve in postoral sucrose stimulation of VTA DA neurons and lever press performance was indicated by the results of two experiments. First, selective surgical transection of the common hepatic branch blocked IG sucrose activation of VTA DA neurons. Second, common hepatic branch vagotomy attenuated lever pressing for IG sucrose infusions, although the lack of a complete blockade of lever pressing for IG sucrose implicates other vagal or non-vagal pathways in this response. Nevertheless, the authors implied that the results are consistent with the finding of normal sugar-conditioned flavor preferences in animals with SDV sparing the common hepatic branch [99]. However, IG carbohydrate conditioning was observed in animals with surgical SDV that included the common hepatic branch as well as in animals with selective common hepatic branch vagotomy [97–99, 102]. A potential role of dorsal root afferents innervating the hepatic-portal vein and projecting via the celiac/superior mesenteric ganglia and splanchnic nerve to the spinal cord in mediating the effects of absorbed glucose on the hypothalamus is indicated by the findings of Goldstein et al [103], but it is not clear whether this pathway is involved in the learning process.

To summarize, advances in selective neural manipulation and recording have significantly contributed to progress in understanding the nature of the unconditioned sugar signal generated in the gut and the potential pathways linking this signal to reward and reinforcement behavior in the brain. One common finding relates to the importance of intestinal SGLT1 sensing to glucose-conditioned preferences. Recent studies indicate that hepatic-portal glucose also contributes to preference learning, although how the sugar is sensed and signaled to the brain is not certain. Also unknown is the mechanism by which postoral fructose conditions flavor preferences in some inbred mice (e.g., FVB/N) [88].

## Mechanisms for fat-conditioned preferences

As in the case of carbohydrates, many studies demonstrated that orally consumed or postorally infused fat emulsions condition flavor preferences, including that of fat, in rats and mice [27, 85]. Flavor preferences vary as a function of fat source, with long-chain triglycerides being more effective than medium-chain triglycerides, and some triglyceride fat sources more effective than others (e.g., corn oil and safflower oil vs. beef tallow and vegetable shortening) [104]. In rats, postoral fat infusions condition weaker flavor preferences than do isocaloric sugar infusions [105] and require more training trials [106], but this is not the case in mice [107–109].

In addition to conditioning CS+ flavor preferences, IG fat infusions rapidly stimulate CS+ intakes in the first training sessions in mice, which suggests a preabsorptive site of action [107, 109]. In order to be effective, infused fat must be digested to fatty acids which can act on multiple intestinal fatty acid sensors including CD36, GPR120 [O3FAR1], and GPR40 [FFAR1] [110] (Fig. 2). CD36 KO mice did not differ from WT mice in their preference conditioning response to IG soybean oil infusions [111]. In contrast, GPR40/120 double knockout (DKO) mice showed only a marginal fat-conditioned flavor preference with 1-h training sessions relative to WT mice (58% vs. 81%) [112]. However, with 24-h training, GPR40/120 DKO mice displayed a more substantial conditioned preference although still weaker than that of WT mice (70% vs. 96%). The 24-h results indicate that other intestinal or postabsorptive sensors contribute to long-term fat-conditioned preferences, e.g., GPR41, GPR43, GPR119 [34].

The gut–brain pathways that mediate postoral fat conditioning are not fully understood. Early studies indicated that vagal afferents are not essential because surgical or capsaicin vagal deafferentation did not prevent animals from learning to prefer a CS+ flavor paired with postoral fat infusions [98, 113]. However, Qu et al. [97] reported that, unlike control mice, SDV mice did not learn to prefer an orally consumed 7.5% fat emulsion over a 30% emulsion, which was taken as evidence for “a deficit in lipid postoral signaling.” Why control mice preferred the less concentrated emulsion was not explained but it may have occurred because the satiating actions of the 30% fat counteracted its postoral appetition actions [114]. Conceivably, the SDV mice did not come to prefer the 7.5% fat because vagotomy reduced the satiating and therefore the appetition-limiting actions of the 30% fat. In another study by the same investigators [84], bilateral afferent vagotomies were produced by targeting nodose neurons using the neurotoxin caspase (Caspase vagotomy) or CCK receptor-expressing vagal neurons using the neurotoxin saporin (CCK-SAP vagotomy). Flavor conditioning was evaluated by training mice (1-h/day) with a CS+ flavor paired with IG infusions of 5% lipid and a different CS+ flavor paired with IG infusions of 20% lipid (which were diluted in the gut to 2.5% and 10% lipid, respectively by the consumed CS solutions). With this procedure, the control mice learned to prefer the CS+ 20% flavor to the CS+ 5% flavor while the sensory vagotomized mice equally preferred the two CS+ flavors. This finding, however, does not demonstrate that the vagotomized mice were completely insensitive to postoral fat reinforcement because their preference for a water-paired CS− flavor was not evaluated [115]. Also, the control and vagotomized groups displayed similar increases in CS+ 5% and CS+ 20% intakes during one-bottle training sessions which is indicative of postoral fat reinforcement [116]. On the other hand, in operant licking tests reinforced with IG infusions of 20% fat, Caspase and CCK-SAP vagotomized mice, unlike controls, did not increase their licking responses over test sessions which indicates a reduced sensitivity to postoral fat reinforcement [84].

In addition to investigating postoral fat reward, Han et al. [84] reported on the reward effects of optogenetic activation of vagal afferent neurons projecting to the upper gut, using a combination of a Cre-expressing adeno-associated virus injected into the stomach and duodenum retrogradely transported to the nodose ganglia, and a Cre-dependent light-sensitive depolarizing channel injected into the left or right nodose ganglia. Using this approach, they demonstrated that optogenetic activation of gut-projecting afferent neurons in the right nodose ganglion (NG) had rewarding actions as indicated by reinforcing (a) nose poking behavior; (b) place preference conditioning; (c) flavor preference conditioning; and by stimulating (d) dorsal striatal dopamine release. In contrast, activation of neurons in the left NG had none of these effects. The optogenetic findings imply that the right nodose mediates fat-conditioned preferences, although Han et al. [84] did not evaluate the effects of unilateral vagal afferent silencing on fat conditioning. The failure of left NG activation to have reinforcing effects implies that vagal afferents mediating sugar reward do not pass through the left NG, but Tan et al. [65] reported that intestinal glucose equally activates vagal neurons in the left and right NG. Further research is needed to resolve the vagal pathways involved in fat and sugar reward.

The finding that selective deactivation of CCK-responsive vagal afferents blocks flavor conditioning suggests a possible role of nutrient-stimulated CCK release in such conditioning. An early study reported that pairing a CS+ flavor with systemic injection of a low dose of exogenous CCK conditioned a mild flavor preference while higher doses were ineffective or conditioned a flavor avoidance [117]. Yet, blocking CCK receptors with devazepide did not prevent IG nutrient-conditioned preferences, indicating that CCK signaling is not essential for postoral nutrient conditioning [118]. Ghrelin is another gut hormone implicated in food reward processing, but experiments with ghrelin receptor KO mice and ghrelin receptor antagonists indicate that ghrelin signaling is not essential for flavor preferences conditioning by IG sugar or fat infusions [77].

In summary, contrary to earlier surgical vagotomy results, recent findings implicate vagal afferents perhaps limited to the right nodose ganglion in flavor conditioning by dilute vs. concentrated fat emulsions and in operant licking for IG fat infusions [84]. Additional work is needed to verify the exclusive involvement of vagal afferents on the right side in CS+ high vs. CS+ low fat conditioning as well as fat-conditioned CS+ preferences relative to a water-paired CS−.

## Mechanisms for protein-conditioned preferences

Orally consumed or postorally administered dietary proteins condition flavor preferences in animals [27, 85]. Relatively little is known, however, about the postoral mechanisms mediating this form of nutrient learning. In rats protein-conditioned flavor preferences are differentially altered by postoral carbohydrate and protein loads, indicating that the animals distinguish between postoral signals generated by these nutrients [119]. Given the diversity of proteins, it is likely that postoral signaling is mediated by one or more common amino acids. Glutamate is one such amino acid and is the prototype for the umami taste receptor (T1R1+T1R3) found in oral taste buds and intestinal enteroendocrine cells [120]. IG infusion of monosodium glutamate (MSG) conditions CS+ flavor preferences in rats and mice [121–123]. Total subdiaphragmatic vagotomy (SDV) and SDV with spared hepatic branch blocked flavor conditioning by IG MSG infusions whereas selective common hepatic branch vagotomy was ineffective [100]. SDV also greatly reduced the activation of brain areas by IG MSG infusions [100]. These findings implicate vagal afferents outside the common hepatic branch in postoral glutamate reinforcement, although this requires confirmation with more selective vagal deafferentation procedures. The postoral glutamate sensor that mediated MSG conditioning is not known but does not require the T1R3 receptor. This is indicated by the finding that T1R3 KO mice, like WT mice, develop preferences for MSG and a MSG-paired CS+ flavor after one-bottle training [124]. The role of other gut glutamate sensors (mGlu1, mGlu4, CaSR) in MSG conditioning remains to be investigated [120].

Thus, there is now evidence implicating vagal afferents in the appetite (preference and acceptance) conditioning actions of sugar, fat, and glutamate in the gut. Interestingly, other recent findings implicate vagal afferents in the hunger state induced by fasting [125, 126]. In one study, selective ghrelin receptor (GHSR) knockdown in vagal afferent neurons abrogated the hyperphagic effect of ghrelin administered at dark onset and caused other behavioral and metabolic impairments [126]. Another study identified a subpopulation of fasting-activated NTS neurons co-expressing epinephrine and NPY, the optogenetic activation of which stimulated feeding and generated conditioned place preference [125]. This is in marked contrast to the conditioned place preference produced by activation of vagal afferents linked to postoral fat reward [84]. Taken together, these findings indicate that distinct vagal-NTS pathways mediate the appetite/reward actions of nutrients in the gut and the hunger/aversive actions of fasting.

## Implications for food choice behavior and treatment or prevention of obesity

From the above discussions, it is clear that rodents use signals generated by the interaction of specific nutrients with upper intestinal enteroendocrine/neuropod cells and vagal sensory neurons to learn preferences and make choices. There seem to be separate signals for acceleration (appetition, reward) and deceleration (satiation) of intake, and the combined effects are important determinants of total energy intake at least in the short term. However, because in most studies, relatively simple binary choices such as glucose vs. water, or low vs. high concentrations of fat emulsions were used [but see [127]], translation to real world situations with much more complex food choices is difficult. As discussed elsewhere, nutrient-conditioned preferences are documented in humans, but such conditioning is less readily obtained in humans, particularly adults, than in rodents [27, 128, 129]. Future studies need to address these species differences. We also have not yet seen any study that examines macronutrient choice behavior in rodents with specific pathway deletions. For example, would permanent silencing of the neuropod signal which renders mice unable to recognize glucose [68] change their long-term macronutrient choice using the geometric model?

Another unanswered question is whether the changes in energy intake resulting from specific pathway deletions have any long-lasting effects on energy regulation and the development of obesity, as quantitative or qualitative changes in food intake do not necessarily lead to changes in body weight. For example, would silencing glucose-sensitive NTS neurons which suppress 24-h sugar intake [65] lead to long-term reductions in the intake of sugar-rich drinks or foods and thereby attenuate sugar-induced obesity? Tools are now available to carry out inducible deletions of specific populations of vagal afferent neurons. Given that at least some pathways include vagal afferent signaling, could it be that the numbing of vagal afferent function observed in high-fat fed mice [130] includes these critical vagal afferent populations, and what implications might this have on the course of obesity?

Bariatric surgeries are currently the most effective treatment option for obesity, and there is great interest in deciphering the mechanisms for their success. A role for vagal afferents contained within the celiac branches in the weight loss effects of Roux-en-Y gastric bypass surgery has been demonstrated in rats [131], but it is not known whether the surgical celiac branch vagotomy affected the vagal afferents mediating the neuropod signal described by Buchanan et al. [68] or the vagal afferent neurons described by Tan et al. [65]. In another study in rats, the common hepatic branch of the left vagus, which was implicated in the detection of the sugar signal by Fernandes et al. [83], was not required for the weight-lowering effects of RYGB [132]. It was claimed that a gut-vagal afferent-striatal pathway is recruited by RYGB to reduce fat appetite in obese rats [133]. However, when this pathway was interrupted with total subdiaphragmatic truncal vagotomy (SDV), RYGB reduced body weight to exactly the same extent as in sham vagotomized rats [133]. Again, the discrepancy in these outcomes could be due to the issues with non-selectivity of SDV and common hepatic branch vagotomy that were described above.

As to potential relevance of sugar-conditioned preferences for treating or preventing obesity, it may be feasible to mimic the absorption of sugar by activating the downstream signaling pathways. Tan and colleagues have already provided proof of principle for such an approach by injecting a Cre-dependent AAV encoding an excitatory designer receptor into the proenkephalin-expressing neurons in the cNTS that are critical for preferences based on sugar sensing [65]. Activating the designer receptor led to a complete switch in licking from a preferred sweet grape-flavored solution to a previously much less preferred non-sweet cherry-flavored solution [65]. It would be interesting to test whether other components of the gut-to-brain sugar-signaling pathway, such as the specific vagal afferent neuron population, or the molecular pathways coupling the SGLT1 transporter to vagal afferents could also be co-opted. Once the specific signaling pathways for fat and protein preference have been identified they could be similarly co-opted for healthier eating.

## Conclusions and future directions

The wide availability of foods rich in sugar and fat is a significant factor in the current obesity epidemic. The inherently attractive flavor of these foods is one factor that promotes their selection and consumption. Rodent studies have established that sugar and fat also activate nutrient sensors in the gut that signal brain reward and learning systems that further enhance the wanting and liking of foods high in these nutrients. Until recently, little was known about the gut–brain pathways that transmit nutrient-generated appetition signals. Recent studies now implicate vagal afferent connections between intestinal nutrient sensing enteroendocrine and neuropod cells and caudal NTS neurons which project to higher brain systems. In the case of sugars, glucose binds to the SGLT1 transporter/sensor on neuropod cells which, in turn, activates glutamatergic synaptic receptors on adjacent vagal afferent fibers. Postabsorptive glucose is also detected at hepatic-portal sites although the sensing mechanism and signaling pathway to the brain are uncertain. In the case of fats, fatty acids act in part on GPR40 and GPR120 intestinal receptors which, in turn, stimulate CCK-sensitive afferent fibers. Other pre- and/or postabsorptive fatty acid sensors are also implicated in postoral fat appetition. Central neural systems triggered by these visceral appetition signals include striatal dopamine circuits and limbic motivational and hippocampal memory circuits [134, 135]. Many details remain to be elucidated, including the relative ineffectiveness of some sugars (fructose, galactose) to stimulate appetite, failure of surgical vagotomy to block flavor conditioning, and the contribution of visceral appetition signals to long-term food intake and body weight regulation. Most importantly, the role of the newly revealed gut nutrient sensors and gut–brain pathways in human food appetite and preferences, and how these gut appetition mechanisms might contribute to therapeutic approaches to overeating and obesity, need further exploration.
